# The Role of TRAIL/DRs in the Modulation of Immune Cells and Responses

**DOI:** 10.3390/cancers11101469

**Published:** 2019-09-30

**Authors:** Duygu Sag, Zeynep Ozge Ayyildiz, Sinem Gunalp, Gerhard Wingender

**Affiliations:** 1 Izmir Biomedicine and Genome Center (IBG), 35340 Balcova/Izmir, Turkey; 2 Department of Medical Biology, Faculty of Medicine, Dokuz Eylul University, 35340 Balcova/Izmir, Turkey; 3 Department of Genome Sciences and Molecular Biotechnology, Izmir International Biomedicine and Genome Institute, Dokuz Eylul University, 35340 Balcova/Izmir, Turkey; zeynepozge.ayyildiz@ogr.deu.edu.tr (Z.O.A.);; 4 Department of Biomedicine and Health Technologies, Izmir International Biomedicine and Genome Institute, Dokuz Eylul University, 35340 Balcova/Izmir, Turkey

**Keywords:** apoptosis, immune regulation, tumor, myeloid cells, lymphoid cells

## Abstract

Expression of TRAIL (tumor necrosis factor–related apoptosis–inducing ligand) by immune cells can lead to the induction of apoptosis in tumor cells. However, it becomes increasingly clear that the interaction of TRAIL and its death receptors (DRs) can also directly impact immune cells and influence immune responses. Here, we review what is known about the role of TRAIL/DRs in immune cells and immune responses in general and in the tumor microenvironment in particular.

## 1. Introduction

The ‘Tumor Necrosis Factor-Related Apoptosis Inducing Ligand’ (TRAIL, CD253, TNFSF10) is a member of the ‘Tumor Necrosis Factor Superfamily’ (TNFSF), along with other closely related ligands, like TNF and CD178 (FasL, CD95L) [1,2,3,4]. Although, TRAIL is a type II transmembrane protein, protease cleavage at the membrane can generate a soluble version. In humans, five receptors for TRAIL are known. DR4 (CD261, TRAIL-R1, TNFRSF10A) and DR5 (CD262, TRAIL-R2, TNFRSF10B) contain a functional intracellular death domain (DD) required for signaling. Depending on the particular signaling pathway utilized, these signals can lead to three different outcomes [1,2,4]. On the one hand, cell death can be induced either via caspase 8- and caspase 3- dependent apoptosis or by necroptosis in a caspase-independent manner. On the other hand, DR4/DR5 signals can support survival, cell migration, and proliferation. Details on the respective signaling pathways have been reviewed previously [2,5,6,7]. The other three receptors described in humans lack a functional death domain and are, therefore, considered decoy receptors. These include the membrane-bound DcR1 (CD263, TRAIL-R3, TNFRSF10C) and DcR2 (CD264, TRAIL-R4, TNFRSF10D), and the soluble osteoprotegerin (OPG, TRAIL-R5, TNFRSF11B), which also can bind RANKL (TNFSF11) [8]. In contrast to humans, mice only express three receptors, DR5, DcR1 (Tnfrsf23), and DcR2 (Tnfrsf22) [1,4].

Early work indicated that particular tumor cells are highly susceptible towards TRAIL-induced apoptosis, with little harm to healthy cells [1,2,3,4]. Due to its great potential as anti-tumor drug, much work on TRAIL and its receptors has been and still is devoted on its development for anti-tumor therapy [1,4,9,10]. In addition to the cancer cells and their surrounding stroma, the tumor microenvironment contains innate and adaptive immune cells that can recognize and destroy tumors. TRAIL expression by immune cells is one major mean by which immune cells can induce apoptosis of tumor cells. However, it becomes increasingly clear that TRAIL/DRs interaction can also directly impact the function of immune cells in many ways. In this review, we will first outline what role TRAIL and its receptors can play in immune cells in general (Section 2) and will discuss some open questions (Section 3). Then, we will outline how these and other findings are relevant for the anti-tumor responses (Section 4).

## 2. Expression and Function of TRAIL/DRs in Immune Cells

### 2.1. Myeloid Cells

#### 2.1.1. Neutrophils

Human blood-derived neutrophils constitutively express mRNA for TRAIL [11,12,13]. However, how much of the TRAIL can be found on the cell surface, if any at all, seems to depend on the donor [11,12,13]. It was suggested that TRAIL is rather pre-stored intracellular in granules [14,15,16] and that some of this pre-stored TRAIL is in the cleaved soluble form which could facilitate secretion [14,16]. Indeed, upon activation, human neutrophils upregulate TRAIL and secrete functional soluble TRAIL. This was observed most potently with IFNα and IFNβ [14,15,17,18], but also, to a lesser extent [13], with IFNγ [11,13,15]. Several other pro-inflammatory stimuli, like fMLP, IL-8, Hsp96 [15]; IL-17 [19]; the CD184-ligand SDF1 [20]; and the TLR2-ligand Pam3C [14]; were shown to boost the TRAIL-release by human neutrophils. In contrast, no impact on the TRAIL expression of human neutrophils was reported for IL-1, G-CSF, GM-CSF, TGFβ, and the TLR3-ligand Poly I:C [11,13,14,17]. Conflicting data were reported for TNF and the TLR4-ligand LPS, with some reports finding either a boost of TRAIL on neutrophils [14,15,18], no effect [13,17], or even a down-regulation of TRAIL [11]. In mice, TRAIL is not expressed by neutrophils [21,22], but can be upregulated following IFNβ stimulation [23]. Interestingly, stimulation of mouse neutrophils with IL-6 and G-CSF induced a tumor-promoting N2 phenotype, characterized by TRAIL down-regulation [24,25], but TRAIL expression could be rescued by IFNγ or TNF [26].

Most studies reported expression of DR5 [11,12,17,20] and DcR1 [11,12,20,27,28,29] on human neutrophils, and only two reports show DR4 and DcR2 expression [20,29]. Importantly, the expression of DR4/DR5 on neutrophils tended to be lower than the expression of the decoy receptors DcR1/DcR2 [11,12,17,28,29]. Activation of human neutrophils with TNF or SDF1 [11,20] or of mouse neutrophils with IFNβ [23] increased the expression of DR4/DR5. In contrast, TNF stimulation [11] or ER stress [30] down-regulated DcR1/DcR2 on human neutrophils. In mice, the expression of DR5 was observed on neutrophils [28,31,32,33], but DcR1 and DcR2 were not detected in the one study that tested their expression [34].

In line with the expression of death receptors, most [20,23,28,29] but not all [17] studies indicated that freshly isolated neutrophils are not sensitive towards TRAIL-induced apoptosis. However, the neutrophils became sensitive following activation [11,20,23,29] or after aging [12,20]. Interestingly, upon such aging, senescent neutrophils upregulated CD184/CXCR4 [35] and became receptive to SDF1, which increased TRAIL-sensitivity, via upregulation of DR4 and DR5 [20]. This aided the migration of the senescent neutrophils to the bone marrow for apoptotic removal in the mouse model [20].

Functionally, most reports are in line with the interpretation that TRAIL/DR-activity is involved in the apoptotic removal of activated, stressed, or aged neutrophils in vivo. As neutrophils are major drivers of inflammation [36,37,38], their elimination usually limits the inflammation and the resulting tissue damage and promotes the resolution of the inflammation. Therefore, TRAIL-deficient mice displayed reduced neutrophil apoptosis, leading to increased neutrophil numbers and inflammation. This was noted during TLR-ligands induced sepsis [39], in the bleomycin model of lung fibrosis [21], and following *Streptococcus pneumoniae* infection of the CNS [31]. Consequently, the administration of soluble TRAIL or agonistic αDR5-antibodies increased neutrophil apoptosis, leading to ameliorated inflammation following *Streptococcus pneumoniae* infection [31] and during sepsis induced by bacteria [32,34] or TLR-ligands [39]. It was also observed in other systems that blocking of neutrophil apoptosis augments inflammation and tissue damage [40]. However, the TRAIL-sensitivity of activated neutrophils was not seen in all models, as, for examples, following *S. pneumoniae* infection of the lung neutrophil-apoptosis was unaffected by the absence of TRAIL [41].

Additionally, the TRAIL produced by activated human neutrophils themselves could mediate cytotoxicity of TRAIL-sensitive tumors [13,14,17,42,43]. However, blood-derived human neutrophils of some tumor patients (squamous cell carcinoma [44]; B cell chronic lymphocytic leukemia [45]) expressed less TRAIL than healthy donors and IFNα-therapy in vivo enhanced TRAIL expression on neutrophils of chronic myeloid leukemia (CML) patients [18]. Besides tumor cytotoxicity, neutrophil-derived TRAIL was also shown to be involved in the resolution of inflammations by targeting macrophages. Neutrophil-derived TRAIL could induce apoptosis of alveolar and lung macrophages in *S. pneumoniae* infected mice [41]. This apoptosis of *S. pneumoniae*-infected macrophages supported the bacterial clearance in the airways and limited the inflammation and the ensuing tissue damage [41].

#### 2.1.2. Monocytes and Macrophages

Freshly purified human blood monocytes have a basal level of intracellular TRAIL expression [11,46]. Conflicting data were reported for the surface expression of TRAIL on human blood monocytes; with two studies reporting no TRAIL-expression [46,47] and two studies showing expression [11,48]. In vitro generated human monocyte-derived macrophages express high levels of TRAIL intracellularly, but low levels on the surface [48]. TRAIL expression can be rapidly induced in human monocytes via IFNα [47,49,50,51,52] and IFNβ [53]. Furthermore, secretion of soluble TRAIL after IFNα stimulation has been reported for human monocytes [17]. In addition, installation of the IFNα-inducer BCG into the bladder of bladder cancer patients led to an increase in TRAIL expression on tumor macrophages [54]. For the treatment of human blood monocytes with LPS, increased TRAIL surface expression was detected after 24 h incubation [48], but not after 12 h [47]. Apart from that, PEDF, an anti-angiogenic and anti-inflammatory agent widely used in clinical trials for cancer treatment [55], induces TRAIL production by human monocyte-derived macrophages [56]. In contrast, exposure of human CD14^+^ monocytes to C-reactive protein (CRP) led to a down-regulation of TRAIL [57]. In mice, approx. 25% of peritoneal macrophages express TRAIL [58]. TLR2 (lipoteichoic acid), TLR3 (poly I:C) and TLR4 (LPS) ligands, but not a TLR9 ligand (CpG), induced TRAIL expression in murine macrophages [59]. Moreover, murine bone-marrow derived macrophages (BMDMs) treated with PEDF in vitro or tumor infiltrating macrophages from PEDF-treated mice upregulated TRAIL expression [56]. Chemically induced ER stress also led to upregulation of soluble TRAIL in a murine monocytic cell line and in primary mouse peritoneal macrophages [60].

Human peripheral monocytes and monocyte-derived macrophages express functional DR4 and DR5, with DR5 being expressed usually higher than DR4 [28,47]. In contrast, tissue resident macrophages have very low DR5 expression [28]. Although one study demonstrated surface expression of DcR1 in human peripheral monocytes [47], other studies reported that decoy receptors are barely expressed by monocytes [28,52]. Macrophages tend to express higher level of DcR1 compared to monocytes [28]. However, macrophages are heterogeneous with several subpopulations displaying different functional activities. Macrophages can differentiate into two main types in the presence of certain polarization factors when recruited into peripheral tissues [61]. Pro-inflammatory signals, like LPS and IFNγ treatment, polarize macrophages towards M1 or classically activated macrophages, that are cytotoxic, pro-inflammatory, and are potent in fighting tumors and pathogens. In contrast, anti-inflammatory cytokines, like IL-4, IL-10, and IL-13, induce M2 or alternatively activated macrophage, that play a role in immune suppression, tumor promotion, angiogenesis, and tissue remodeling [61]. Death receptors are differentially expressed on M1 and M2 type macrophages. M2-like tumor associated macrophages (TAMs) and M2 polarized THP-1 macrophages express more DR5 than M1 macrophages [28,62,63]. Consistently, agents that support M2 polarization enhance DR4 and DR5 expression on human monocytes and monocyte-derived macrophages [28]. Furthermore, inflammatory mediators such as IFNγ [47] and IFNα [52] can downregulate surface expression of DR5 on human blood monocytes. In contrast, one study reported that LPS augments DR4 expression on human monocytes [64]. Furthermore, M1 macrophages in the synovium of rheumatoid arthritis patients expressed higher levels of DR5 than M2 macrophages [65]. In mice, monocytes and macrophages express DR5 constitutively [28,62,65,66,67,68] and its expression can be augmented by the DNA-binder trabectedin [62]. 

Peripheral human monocytes are sensitive to TRAIL-induced apoptosis with 50% reduction in their survival 72 h after TRAIL treatment in vitro [28]. Furthermore, pre-treatment with the anti-inflammatory cytokines IL-4 or IL-10 seemed to sensitize monocytes towards TRAIL-mediated cell death [28,69]. TRAIL also induced apoptosis in the human macrophage cell line U937 [70] and in murine peritoneal macrophages [71]. M2 macrophages are more sensitive to TRAIL-induced apoptosis than M1 macrophages due to their higher DR5 surface expression [28] and due to the increased O-glycosylation of DR5, which augments receptor oligomerization [63]. Sensitivity of monocytes and macrophages to TRAIL-mediated apoptosis was also reported in disease models. In a mouse tumor model, TRAIL treatment in vivo was shown to suppress tumor growth by the elimination of monocytes and M2-like TAMs in the tumor [28,62]. In the atherosclerosis ApoE^−/−^ mouse model, TRAIL treatment in vivo selectively induced apoptosis in vascular infiltrating inflammatory macrophages [66,67]. This may be due to the upregulation of DR5 on vascular macrophages by ER stress in advanced stages of atherogenesis [72]. In general, activated macrophages are sensitive to TRAIL-induced apoptosis and this is crucial for the regulation of homeostasis [58,71]. Besides inducing apoptosis, TRAIL-mediated signals can make monocytes and macrophages also anti-tumorigenic [17,47,50,52,53,73].

Apart from apoptotic effects, TRAIL/DR interaction can also impact monocytes and macrophages in other ways. For instance, TRAIL-treatment can enhance monocytic maturation of primary human CD34^+^ hematopoietic stem cells without inducing any cytotoxicity [74]. Furthermore, TRAIL-mediated DR4-triggering could induce migration of THP-1 monocytes and of LPS-activated primary human monocytes [64]. Moreover, treatment with soluble TRAIL induced the expression of pro-inflammatory cytokines in human monocyte-derived macrophages and in mouse peritoneal macrophages [75]. Similarly, in tumor-challenged nude mice injected with soluble TRAIL, TAMs were reported to exhibit increased expression of pro-inflammatory cytokines [75]. In contrast to these pro-inflammatory effects of TRAIL, one study reported an anti-inflammatory role. In a mice model of colitis-associated colorectal cancer (CAC), early treatment of TRAIL in vivo protected the mice by reducing the inflammation, probably by decreasing the levels of infiltrating macrophages and pro-inflammatory cytokines and increasing the percentage of M2 macrophages [76]. Additionally, after stimulation with LPS and TRAIL in vitro, the murine RAW264.7 macrophage cell line displayed a reduction in iNOS, IL-6, and TNF expression compared to LPS alone [76].

#### 2.1.3. Dendritic Cells

Human dendritic cells (DCs), either isolated from blood or monocyte-derived, expressed no surface TRAIL in most [47,49,77,78,79,80] but not all [46,81] studies. However, TRAIL could be detected intracellular [46,77,78,79,80] and in the culture supernatant [79]. Similar to neutrophils, the activation of DCs by IFNα [49,77,82,83,84,85,86,87,88] or IFNβ [77,81,85] increases the levels of membrane-bound and soluble TRAIL. Known inducers of IFNα/IFNβ, like TLR-ligands [77,84,88,89,90] and virus particles [85,88,91,92,93], also induce TRAIL expression/secretion by human DCs. Conflicting results obtained with LPS could be due to the timing, as TRAIL induction was not observed after 12 h [49,81] but after 1-2 days [82,90] of incubation. Disagreement also exists about the impact of IFNγ on the TRAIL expression on human DCs [49,85]. Interestingly, it was suggested that CD40-ligation [77] or the action of regulatory T cells (Tregs) [94] could inhibit the upregulation of TRAIL on stimulated human DCs. Similar to humans, in mice, TRAIL was found to be upregulated on mouse DCs by IFNα and IFNβ directly [86] or indirectly [94,95,96]. Furthermore, IL-15 was suggested to be an inducer of TRAIL expression on mouse DCs [97,98].

With the exception of one report showing a weak staining for surface DR5 [90], most studies did not detect surface expression of DR4/DR5 [46,90,99,100] or DcR1/DcR2 [90,100] on human DCs. However, DR4 and DR5 could be detected by mRNA [82,99] and intracellular staining [46]. The surface expression of DR4 and DR5 was upregulated after 2 days of stimulation with LPS in vitro [82]. However, in another study the incubation with LPS for 20 h resulted in a different outcome, with DR4 being unchanged and DR5 being down-regulated [90]. In the mouse system, DR5 surface expression was reported for bone-marrow derived DCs [101].

Surprisingly, little is known about the sensitivity of DCs towards TRAIL-induced apoptosis. Two studies, one on human monocyte-derived DCs [90] and one on mouse bone-marrow derived DCs [101], suggest that stimulated DCs are less sensitive towards TRAIL-induced apoptosis than immature DCs. 

In contrast, many studies demonstrated that human DCs can exert TRAIL-dependent cytotoxic activity against various tumors cells. This was reported for unstimulated DCs [46,102], for IFNα/β stimulated monocyte-derived DCs [49,77,81,82,83,87,92,103,104], for pDCs [78,79], and for DCs stimulated with TLR-ligands [77,84,88] or virus particles [85,88,91,93,105]. Similar observations were made in the mouse system [86,95,96,98]. Interestingly, two studies suggest that TRAIL/DR interaction can also have a direct impact on the TRAIL^+^ DCs in an apoptosis-independent manner, although, they conflict in their implication. For human monocyte-derived DCs stimulated with LPS, TRAIL acted like a co-stimulatory molecule, as blocking TRAIL reduced the upregulation of activation markers and the production of cytokines by the DCs [90]. In contrast, in a mouse study, the engagement of DR5 on mouse DCs impaired the antigen-presenting functions of the DCs, leading to reduced priming of CD4^+^ and CD8^+^ T cells [106]. 

#### 2.1.4. Other Myeloid Cells

##### Basophils and Mast Cells

Primary human blood-derived basophils expressed DR4 and DR5 in most but not all donors [107,108] and were resistant towards TRAIL-induced apoptosis [107]. In contrast, neoplastic basophils from chronic myeloid leukemia (CML) patients were TRAIL sensitive, despite lower expression of DR5 [107]. Human cord blood-derived mast cells (CBMCs) were reported to express only DR5 on the cell surface, although mRNA for DR4, DcR1, and DcR2 was detected in most of the donors [109]. These CBMCs were also sensitive towards TRAIL-induced apoptosis [109,110]. In contrast, primary human lung mast cells were reported to be negative for surface DR4 or DR5 expression [108].

##### Eosinophils

Human blood-derived eosinophils did not express surface TRAIL [111], but eosinophils upregulated TRAIL during inflammatory responses [111,112,113]. Eosinophils can express all four death receptors (DR4, DR5, DcR1, and DcR2), although the expression levels reported varied [29,113,114]. Interestingly, during inflammation, human eosinophils down-regulated the expression of the pro-apoptotic receptors (DR4, DR5) and up-regulated the expression of the decoy receptors (DcR1, DcR2). This was observed in asthmatic patients [113,114] and in patients with parasitic infections [114] or with Churg-Strauss syndrome, a disease characterized by eosinophilia [115]. Importantly, treatment with soluble TRAIL did not induce apoptosis in human eosinophils, but rather increased cell survival [29,113], although variability between donors was noted [29]. TRAIL was also shown to promote lung eosinophilia in an indirect fashion. Human and mouse bronchial epithelial cells incubated with soluble TRAIL produced CCL20 [116,117]. CCL20 caused the influx of IL-5-producing CD4^+^ T cells into the lung in mice [116] and IL-5 is an essential chemoattractant for eosinophils [118,119]. In line with the role of TRAIL in promoting eosinophilia and acute inflammation are animal studies on allergic asthma [116], rhinoviral infection [120], and eosinophilic esophagitis (EoE) [121]. However, one study probing the role of TRAIL late during the allergic asthma inflammation, suggested a protective role for TRAIL [122]. Furthermore, data on mouse models of chronic airway inflammation yielded conflicting results [123,124]. These findings could suggest that the sensitivity of eosinophils towards TRAIL-induced apoptosis might differ during early and late stages of the inflammation. 

##### Myeloid Derived Suppressor Cells

Inhibitory myeloid derived suppressor cells (MDSCs), either derived from monocytes or granulocytes, are prevalent in the tumor microenvironment [125]. Their recruitment/induction can be promoted in mice by chemokines that tumor cells produce following treatment with soluble TRAIL [126]. Human and mouse MDSCs express DR5 [127] and, curiously, the tumor environment could cause an upregulation of DR5 on mouse MDSCs in vivo [127]. It was suggested that the increase of DR5 on MDSCs is a consequence of ER stress [62,127], which also led to a down-regulation of DcR1 and DcR2 on human MDSCs [127]. Consequently, human and mouse MDSCs are sensitive towards TRAIL-induced apoptosis [127], which was utilized already therapeutically to remove MDSCs in vivo in preclinical [62,127] and clinical settings [30]. However, another study found little DR5 expression on tumor-associated mouse MDSCs [28], suggesting that the TRAIL-sensitivity of MDSCs could vary depending on the tumor studied.

### 2.2. Lymphoid Cells

#### 2.2.1. Conventional Natural Killer Cells and Innate Lymphoid Cells 1

Freshly purified Natural Killer (NK) cells (CD3^neg^ CD56^+^) from human blood, either peripheral blood or umbilical cord blood, did in most reports not express membrane TRAIL [128,129,130]. Although one study suggested that TRAIL could be detected by intracellular staining [130], another one could not even detect expression of TRAIL mRNA in blood-derived NK cells [131], possibly reflecting donor differences. In those reports were a subset of blood-derived NK cells stained for surface TRAIL, it was found exclusively on the CD56^bright^ population [128,132] and large inter-individual variability was noted [132]. In contrast to the blood, most [128,133,134] but not all [135] studies demonstrated surface TRAIL expression on human liver CD56^bright^ NK cells. A similar expression profile was seen in mice, where only a subset of NK cells in the liver, but not in blood, spleen, or lung, constitutively expressed TRAIL [136,137,138]. In recent years it became clear, however, that these TRAIL^+^ CD56^bright^ cells are most likely not conventional NK (cNK) cells, but rather ILC1s, a related subset of innate lymphoid cells (ILCs) [139,140,141]. Indeed, TRAIL expression by ILC1s but not resting cNK cells seems to be a generic feature of these cells in humans [133] and in mice [142,143,144,145,146,147,148,149,150]. Interestingly, the expression of surface TRAIL on mouse cNK/ILC1s, but not on other cells, was dependent on CD335 (NKp46, Ncr1) [151,152,153,154,155] as in cells lacking CD335, TRAIL remained intracellular [151,153]. The distinction between cNK cells (CD11b^+^ CD49a^−^ CD49b^+^ CD186^−^ Eomes^−^ TRAIL^−^) and ILC1s (CD11b^−^ CD49a^+^ CD49b^−^ CD186^+^ Eomes^−^ TRAIL^+^) is further complicated by the recent finding that mouse cNK cells can, under the influence of TGFβ, convert into ILC1-like cells (CD49a^+^ CD49b^+^ TRAIL^+^) [147,156,157]. This is, therefore, another example of the plasticity of innate lymphoid cells [141,158].

Nonetheless, both cNK cells and ILC1s can upregulate the expression of TRAIL following stimulation. This was seen with IL-2 [128,129,135,146,151,159,160,161], IL-15 [151,159,160,161,162], IFNα/β [50,51,131,163,164,165,166], and IFNγ [136,137,167,168,169,170]. Upregulation of TRAIL on cNK/ILC1 was also seen in patients treated with IFNα in vivo within 4–6 h [131,164,165,171,172,173], which negatively correlated with viral titers [131,172]. Given the prominent role of IFNs in anti-viral immune responses, it is not surprising that TRAIL upregulation was also seen following viral infections or stimulation with purified TLR3 or TLR9 ligands [51,148,166,167,168,169,174,175,176,177].

Little information is available on the expression of DRs on cNK/ILC1s. Whereas, resting human blood-derived NK cells were reported to express low levels of surface DcR2 [27], after in vitro activation only expression of DR5 and DcR2 was seen [160]. However, in vitro stimulated human NK cells were not sensitive towards TRAIL-induced apoptosis [160].

NK cells are prototypic cytotoxic cells, utilizing either soluble factors, like TNF, or granzymes and perforin stored in cytotoxic granules, or the death receptors CD178 (FasL, CD95L) and TRAIL to induce apoptosis in target cells [178,179]. The involvement of TRAIL^+^ cNK/ILC1s for anti-tumor response has been reviewed previously [179,180]. Importantly, cNK/ILC1s can also regulate and limit adaptive immune responses [158,181,182] and TRAIL-mediated cytotoxicity is one of several mechanisms to achieve this. In particular, activated but not resting T cells upregulate death receptors (see Section 2.2.2) and become sensitive towards apoptosis induced by TRAIL^+^ cNK/ILC1s. This was observed for CD4^+^ [177,183] and CD8^+^ [176] T cells and appears particularly important in the case of chronic virus infections, like MCMV in mice [177] and hepatitits B virus (HBV) [176]. By this TRAIL/DR-dependent removal of activated, antigen-specific T cells during the chronic MCMV infection, the cNK/ILC1s were able to restrain the T cell responses in mice and to limit tissue damage and the risk for autoimmunity [177]. Another TRAIL/DR-dependent means to limit T cell responses by mouse cNK/ILC1s was the induction of apoptosis in immature but not mature DC in vivo [101]. Interestingly, engaging DR5 on mouse DCs by TRAIL^+^ cNK/ILC1s was also reported to impair antigen-presenting functions of the DCs in an apoptosis-independent manner [106]. 

#### 2.2.2. Conventional αβ T cells

Resting human and mouse T cells do not express TRAIL, but can upregulate it following TCR-mediated activation [58,160,184,185,186,187,188,189,190,191], although, in some reports, TCR-stimulation alone was not sufficient [59,192,193]. In general, this upregulation of TRAIL was stronger on CD4^+^ T cells than on CD8^+^ T cells [50,185,186,187]. Furthermore, in humans, a high degree of variability of TRAIL-upregulation on T cells between different donors was observed. In line with this, the degree of TRAIL-upregulation on mouse T cells was influenced by the genetic background [191]. In most studies, IFNα/IFNβ cytokines alone, similar to NK cells, could induce TRAIL expression on human T cells [50,51,185,186,187] and they could boost TCR-induced TRAIL expression [186,187,192,194].

Expression of all four TRAIL death receptors on naïve human [27,92,183] or mouse [32] T cells appears absent, with one study reporting DcR2 expression on human blood-derived CD8^+^ T cells but not CD4^+^ T cells [27]. Where changes following TCR-triggering were reported, the reports suggest an upregulation of all death receptors, however, at varying degrees [92,160,183,184,190,195]. Interestingly, TCR-mediated stimulation of human T cell lines led to a down-regulation of DR4 and DR5, without changes in DcR1 or DcR2 [184], suggesting that the regulation of the death receptors might differ between primary and secondary responses.

In line with the expression of death receptors, naïve or freshly stimulated human T cells were not sensitive towards TRAIL-induced apoptosis [160,184,196,197]. However, following repeated or chronic stimulation, T cells can become sensitive towards TRAIL-induced apoptosis. One important example for this is the elimination of antigen-specific T cells during viral infections by TRAIL^+^ cNK/ILC1s, as outlined above (see Section 2.2.1), and by TRAIL^+^ pDCs [92]. In a similar fashion, mouse Tregs upregulated membrane-bound TRAIL after TCR-mediated stimulation and induced apoptosis in effector T cells in vitro and in vivo [190]. Another example is the TRAIL-sensitivity of CD8^+^ T cells that have been primed without CD4^+^ T cell help [198,199], mediated by CD27/CD70 interaction [200], although, common γ-chain cytokines, like IL-2, IL-7, or IL-15, could substitute for CD4^+^ T cell help [199,201,202,203]. ‘Helpless’ CD8^+^ T cells expand normally during the primary response, but upon secondary stimulation undergo TRAIL-mediated ‘activation-induced cell death’ (AICD) [198,204]. Such ‘helpless’ CD8^+^ T cells could also release soluble TRAIL and induce fratricide [198] and their TRAIL-expression was implicated in their capability to transfer tolerance (infectious tolerance) [205,206]. Such ‘helpless’ CD8^+^ T cells were also detected in HIV patients with low peripheral CD4^+^ T cell numbers [207]. Interestingly, TRAIL-induced apoptosis also influenced, at least in mice, the balance of Th1- versus Th2-T cells. After TCR-driven in vitro stimulation, mouse Th2 cells expressed TRAIL, but were resistant towards TRAIL-driven apoptosis [191]. In contrast, Th1 cells did not express TRAIL, but were sensitive towards TRAIL-induced apoptosis [191]. Due to this differential sensitivity, TRAIL/DR-engagement could impair Th1 and favor Th2 responses in vitro [191] and in vivo [59,195,208].

Importantly, TRAIL/DR expression on T cells serves also several non-apoptotic functions. The proliferation of human [196,197,209,210] and mouse [195,209,211,212,213,214,215] conventional, naïve T cells following TCR-mediated stimulation was impaired by concomitant TRAIL/DR-engagement. This inhibition was particularly strong with suboptimal TCR-stimulation [197,209] and both DR4 and DR5 were implicated [196,197,211,212]. Such DR-triggered signaling impaired proximal TCR-signals, Ca2^+^ influx, cell cycle progression, and subsequent cytokine production [196,197,209,212,213]. In contrast to naïve T cells, the proliferation of activated mouse CD4^+^ CD25^+^ Tregs was enhanced by TRAIL/DR-engagement in vitro and in vivo [195,216,217]. 

#### 2.2.3. Innate-like T cells

##### Invariant Natural Killer T cells

Expression of functional TRAIL on human [218] and mouse [137,219,220] invariant Natural Killer T (*i*NKT) cells could be induced by antigenic stimulation, although IFNγ was suggested to be involved as well [137]. *i*NKT cells were discovered and studied extensively due to their anti-tumor activity [221,222]. Besides granzyme/perforin- [223,224] and CD178- [225,226] mediated cytotoxicity, TRAIL-dependent anti-tumor responses have been described for human [218,225,227] and mouse [219,228,229] *i*NKT cells.

##### γδ T cells

Expression of membrane bound and soluble TRAIL by human γδ T cells could be induced by TCR-triggering together with IL-2 [230,231,232,233,234] and/or by NKG2D-engagement [235,236]. TRAIL expression on blood-derived IL-17-producing Vγ9Vδ2 T cells was also noted during bacterial meningitis [237]. Furthermore, γδ T cell-agonists could augment serum levels of soluble TRAIL in vivo and this correlated positively with the clinical response in prostate cancer [230] but not in breast cancer [234] patients. Interestingly, the role of TRAIL in the cytotoxicity of the γδ T cells appears to be influenced by the mean by which the target cell is recognized. When the target cells were recognized via NKG2D-engagement, then the cytotoxicity of the γδ T cells was largely dependent on TRAIL [232,235,236]. In contrast, when the target recognition was TCR-driven, then TRAIL played a minor role and the cytotoxicity was largely dependent on perforin [231,233].

#### 2.2.4. B cells

TRAIL expression on resting B cells was not observed in humans [53,238] but in mice on about ^1^/_5th_ of splenic B cells [189]. Upregulation of TRAIL on human B cells was seen with IFNα and IFNα-inducers, like TLR9 ligands [51], but not following stimulation via the BCR, PHA/IL-2, or IFNβ [51,53]. Furthermore, naïve B cells expressed DR4 [27,239,240] and DR5 [32,239,240,241] and their expression levels increased upon stimulation [239,240,242]. Consequently, germinal center (GC) or memory B cells expressed higher levels of DR4 and DR5 than naïve cells [239,240]. However, conflicting data were reported for the expression of DcR1 and DcR2 on B cells [27,239,240].

Whereas naïve B cells are insensitive towards TRAIL-induced apoptosis, they develop sensitivity following activation [239,243]. In line with this, human CD5^+^ B cells [240] and human or mouse plasma cells [243] were reported to be particular sensitive towards TRAIL-induced apoptosis. The lack of protective CD40 ligation on plasma cells was suggested to be the reason for this sensitivity [243]. However, the impact of CD40 appears also dependent on the stage of B cell development or the type of stimulation. On the one hand, CD40 ligation concomitant to IFNα-mediated stimulation could boost TRAIL upregulation on naïve human B cells [51]. On the other hand, it was suggested that CD40 ligation could protect B cells from TRAIL-induced apoptosis early after BCR-mediated activation [240,244] but not 3–5 days later [239]. The connection between CD40 and TRAIL is complicated by two additional aspects. First, the hetero-oligomerization of CD40 and DR5 could dampen the activation of primary human B cells [242]. Second, CD40-ligation on B cells can induce expression of CD25 (IL-2Rα) [245] and IL-2 signaling was reported to cause down-regulation of TRAIL on B cells [246].

Importantly, the TRAIL/DR-interaction could influence the isotype of the antibodies produced by activated B cells. Data from in vivo experiments that either blocked TRAIL/DR-interaction [211,247] or triggered DRs with soluble TRAIL [247] indicated that TRAIL/DR-interaction impairs the production of IgG1 and, to a lesser extent, IgG2a antibodies.

## 3. Common Themes and Open Questions on the Role of TRAIL/DRs in Immune Cells

### 3.1. Regulation of TRAIL-Sensitivity

The sensitivity of immune cells towards TRAIL-induced apoptosis is regulated on several levels.

#### 3.1.1. TRAIL Expression

As outlined above, the expression levels of TRAIL on immune cells can be influenced by intrinsic signals, like ER stress or senescence, as well as many extrinsic signals, like cytokines.

#### 3.1.2. Membrane-Bound vs. Soluble TRAIL

Functional TRAIL can either be membrane-bound or soluble, after cleavage from the surface [1,5]. However, the bioactivity of membrane-bound and soluble TRAIL differs significantly. First, the cytotoxic potential of soluble TRAIL was suggested to be 100–1000-fold lower than that of membrane-bound TRAIL [248]. Second, soluble TRAIL, in contrast to the membrane-bound version, was not able to impair the activation of conventional T cells [197] or to promote the proliferation of Tregs [195]. Mechanistically, it was reported that soluble TRAIL could trigger only DR4, whereas membrane-bound TRAIL could trigger both DR4 and DR5 [249]. Given these distinctions, it is particular important to keep in mind that not all of the TRAIL-bioactivity found in cell culture supernatants or in body fluids stems solely from soluble TRAIL (see Section 3.2).

#### 3.1.3. Expression Levels of the Death Receptors

Similar to TRAIL and as outlined in detail above, the expression levels of the death receptors on immune cells can be influenced by many intrinsic and extrinsic signals. The relative expression of these death receptors is relevant for two reasons. First, it was suggested that the affinity of DR4/DR5 towards TRAIL is higher than that of DcR1/DcR2 [250,251]. Second, it appears that the ratio of functional (DR4, DR5) vs. decoy receptors (DcR1, DcR2) can dictate the sensitivity towards TRAIL. Immune cells could become TRAIL-sensitive by upregulation of DR4/DR5 and/or by the down-regulation of DcR1/DcR2 [11,28,30]. In contrast, an inverse ratio predicts TRAIL-resistance [28,47,90,127]. Although, DR4 and DR5 appear on a first glance redundant, slight functional differences likely allow to fine-tune the response towards TRAIL. Such differences include, for example, the higher affinity of DR5 than DR4 for TRAIL [250,251], the inability of DR5 to bind soluble TRAIL [249], and the ability of DR5 to form hetero-oligomers [242,250]. However, other differences likely remain to be discovered.

#### 3.1.4. Receptor Interactions

Besides the absolute expression levels of death receptors, their activity could also be influenced by the formation of hetero-oligomers. This has been suggested for DR5 and CD40, which reduced CD40-signaling [242], and for DR5 and DcR2, which reduced DR5-signaling [250].

#### 3.1.5. Signaling Pathways

Finally, the outcome of DR-triggering is regulated on the level of the intracellular signaling pathways, which can lead to apoptosis, necroptosis, or increased survival and proliferation. The regulation of these pro- and anti-apoptotic signaling pathways are still poorly understood and are discussed in detail elsewhere [2,5,6,7]. However, it is likely that these pathways contain several potential new drug targets to regulate the resolution phase of immune responses.

### 3.2. TRAIL on Exosomes

Most studies that investigated the TRAIL-activity in cell culture supernatants assumed that the activity is due to soluble TRAIL released from the cells in the culture. However, functional TRAIL could also be detected in exosomes released from various cells, including human T cells [252,253,254], human neutrophils [16], human placental explants [255], mouse bone-marrow-derived DC [256], and from various tumors [257,258,259,260]. Furthermore, TRAIL^+^ exosomes have been detected in sera of tumor patients [257] and in the synovial fluid of arthritis patients [261]. The TRAIL in these exosomes was the full-length, membrane-bound version and not the shorter version of soluble TRAIL [253,257]. Given that the bioactivity of soluble and membrane bound TRAIL are very different (see Section 3.1), it is important to know if the TRAIL-activity in the culture supernatant is due to soluble or exosome-bound TRAIL. However, few studies measured the molecular weight of the TRAIL recovered from the cell culture supernatants. Even transwell-studies could be misleading, as exosomes seem to move freely across pore sizes of 1 µm and can transmit 15–33% of the biological activity across 0.4 µm pores [262,263]. As most cells release exosome [264,265], a reasonable working hypothesis appears to be that all TRAIL^+^ immune cells are able to release TRAIL^+^ exosomes. How this, compared to soluble TRAIL, influences immune responses needs to be addressed in future studies.

### 3.3. TRAIL’s Role in the Resolution of Immune Responses

Looking at the findings outlined so far in a broad sense, it appears that one of the main roles of TRAIL/DRs in the immune system is during the resolution phase of immune responses. By removing senescent, chronically activated, or stressed immune cells at sites of inflammation, TRAIL/DRs regulate innate and adaptive immune responses by terminating the response and by limiting thereby tissue damage and the risk of autoimmunity.

#### 3.3.1. Removing Effector Cells

Activated or senescent neutrophils become sensitive towards TRAIL-induced apoptosis [11,12,20,23,29]. As neutrophils are major drivers of inflammation [36,37,38], their TRAIL-dependent removal supported the resolution of the inflammation [21,23,31,32,34,39,41]. Furthermore, activated T cells are sensitive towards TRAIL-induced apoptosis [176,177,183,190]. Other immune effector cells known to be sensitive towards TRAIL-induced apoptosis are sub-optimal activated, ‘helpless’ CD8^+^ T cells upon secondary stimulation [198,204,207]; terminally differentiated cells, like T cell blasts [210,266]; plasma cells [243]; MDSCs [30,62,127]; and hematopoietic cancers (e.g., [100,188,238]). Activated immune cells greatly increase the synthesis of proteins, which can stress the endoplasmic reticulum (ER), leading to an ‘unfolded protein response’ (UPR) [267,268,269]. Similar, pathogens and chronic cell activation can cause ER stress [267,269,270]. Indeed, it was shown that ER stress increases the TRAIL-sensitivity of macrophages [271] and MDSCs [30,127]. Furthermore, *Streptococcus pneumoniae* infected alveolar macrophages were susceptible towards TRAIL-induced apoptosis [41]. However, beyond these two examples, the link between ER stress and TRAIL-sensitivity is not yet established. The two exceptions in the pattern of TRAIL-induced removal of effector cells, seem to be immature DCs and eosinophils. First, mouse cNK/ILC1s could induce apoptosis in immature but not mature DC in vivo in a TRAIL/DR-dependent manner [101]. Second, the survival and functions of eosinophils were reported to be augmented by TRAIL/DRs [116,120,121]. However, two studies that investigate the role of TRAIL either late during an allergic asthma inflammation [122] or during a chronic airway inflammation [123], suggested that TRAIL now induces apoptosis of eosinophils. These reports might indicate that the impact of TRAIL on eosinophil differs during early and late stages of the inflammation.

#### 3.3.2. Impairing Effector Cells

Besides their direct apoptotic removal of effector cells, TRAIL/DR-activity can also impair the expansion/function of effector cells. Either directly, by impairing the activation and proliferation of pathogenic T cells, or indirectly, by augmenting the proliferation of inhibitory Tregs (see Section 2.2.2).

#### 3.3.3. Limiting Tissue Damage

In line with the idea that the activity of TRAIL/DRs limits ongoing immune response and supports the transition into the resolution phase, is the fact that TRAIL-deficiency or TRAIL/DR-blockage exacerbates, whereas the injection of functional TRAIL ameliorates pathogen burden. This has been noted for *Streptococcus pneumoniae* infection of the CNS [31] or the lung [41], for systemic *Listeria monocytogenes* [33] or MCMV [177] infection, and for influenza vaccination [272] or infection [273]. At first, it might appear counterintuitive to curtail anti-pathogenic immune responses. However, this inhibition is likely aimed at limiting tissue damage. Without an efficient resolution in the absence of TRAIL/DRs, immune responses continue and could become damaging to the host tissue, which eventually could lead to autoimmunity. Indeed, augmented tissue damage and signs of autoimmunity in the absence of TRAIL were observed, for example, following influenza [22], MCMV [177], rhinovirus [120], *Listeria monocytogenes* [33], and *Streptococcus pneumoniae* [31] infections and during sepsis induced by bacteria [32,34] or TLR-ligands [39]. This probably also contributes to the increased susceptibility of TRAIL-deficient mice towards experimental autoimmune diseases, as reported for collagen-induced arthritis (CIA) [274], diabetes [67,274,275], and experimental autoimmune encephalomyelitis (EAE) [195,215].

#### 3.3.4. Avoiding Autoimmunity

The idea that TRAIL/DR-activity limits tissue damage induced by unrestrained immune responses is also supported by the observation that TRAIL/DR-blockage exacerbates, whereas the injection of biologically active TRAIL ameliorates autoimmune diseases. This has been observed for colitis [214], collagen-induced arthritis (CIA) [211,276,277], diabetes [275,278], experimental autoimmune encephalomyelitis (EAE) [215,217,279,280,281], experimental autoimmune thyroiditis (EAT) [208,216], and systemic lupus erythematosus (SLE) [247].

## 4. TRAIL/DRs in the Tumor Microenvironment

### 4.1. Anti-Tumor Cytotoxicity of TRAIL^+^ Immune Cells

Many immune cells express TRAIL constitutively or following activation and thereby can be cytotoxic to TRAIL-sensitive tumor cells in vitro and in vivo. This has been reported for neutrophils [13,14,17,42,43], monocytes/macrophages [17,47,52,73], DCs [46,49,77,78,79,81,82,83,86,87,91,98,102,103,104], pDCs [84,85,88,91,93,95,96,105], cNK/ILC1s [134,136,137,163,228,282], *i*NKT cells [218,219,225,227,229], γδ T cells [231,235], and conventional T cells [186,194,283,284,285,286].

### 4.2. TRAIL Susceptibility of Tumors and Immune-Surveillance

Malignant transformation of cells often leads to sensitivity towards TRAIL-induced apoptosis in a cell-autonomous manner [1,2]. As many activated immune cells express TRAIL, the selective pressure of the anti-tumor immune response forces the evolution of the tumor. This is best illustrated by TRAIL-deficient mice, which are more susceptible towards endogenous tumors developing either spontaneously [287] or induced by the chemical carcinogen methylcholanthrene (MCA) [228]. Furthermore, tumors in TRAIL^−/−^ mice developed metastases more frequently [137,288]. Interestingly, this TRAIL-dependence of metastases was more prominent for some organs, like liver, than others, like lung [137,288], indicating organ differences of immune-surveillance mechanisms. The greater sensitivity towards MCA-induced tumors could also be mimicked by repeated injection of a blocking anti-TRAIL-antibody [289]. Additionally, tumors developing in TRAIL^−/−^ mice retained TRAIL-sensitivity, whereas tumors developing in TRAIL-proficient animals acquired TRAIL-resistance [289]. These reports demonstrate that TRAIL is an important mechanism, besides CD178 (FasL, CD95L) and perforin, in the tumor immune-surveillance. cNK/ILC1s [136,137,228,282,289] and *i*NKT cells [290,291,292] have been suggested to be major players in this tumor immune-surveillance. The presence of TRAIL^+^ ILC1s in the liver [139,140,141] might also explain some of the organ specificity of TRAIL-mediated immune-surveillance [137,288].

### 4.3. Tumor Mechanisms to Evade TRAIL-Mediated Cytotoxicity

The observation that tumors developing in TRAIL^−/−^ but not in wild-type mice retained TRAIL-sensitivity [289] indicates that tumor cells can develop mechanisms to avoid TRAIL-mediated killing [1,2]. The tumor can achieve this by means that are not unique to TRAIL/DRs, like the upregulation of anti-apoptotic molecules [293] or an interference with general aspects of the death-receptor signaling, e.g., by mutating the death domain (DD) [294], by inactivating caspase-8 [295,296], or by overexpressing c-FLIP [297,298]. Several other tumor cell-intrinsic strategies were reported that limit the apoptotic-signals induced by DR4 or DR5 themselves. First, changes in glycosylation can alter the sensitivity of DR4/DR5 [299,300,301,302]. Second, the surface expression of DR4/DR5 can be reduced either by epigenetic changes in the respective genes [303,304], by autophagic removal [305], or by relocation to the nucleus [306]. Third, the tumor can upregulate the decoy receptor DcR1 and DcR2 [1,2,62], which reduces the TRAIL-binding to DR4 and DR5. Consequently, expression of DcR1/DcR2 correlated with poor prognosis for patients with breast cancer [307], prostate carcinoma [308], or acute myeloid leukemia (AML) [309,310]. In contrast, conflicting data have been reported for the correlation between DR4/DR5 expression and patient survival for renal cell carcinoma [311] and hepatocellular carcinoma (HCC) [304]. Furthermore, some single-nucleotide polymorphisms (SNPs) in TRAIL of HCC patients are correlated with overall patient survival [312]. In summary, these reports indicate that TRAIL/DRs are an important player in the anti-tumor response. 

### 4.4. TRAIL/DR-Related Immune-Tumor Cross-Talk in the Tumor Microenvironment

**Figure 1:**
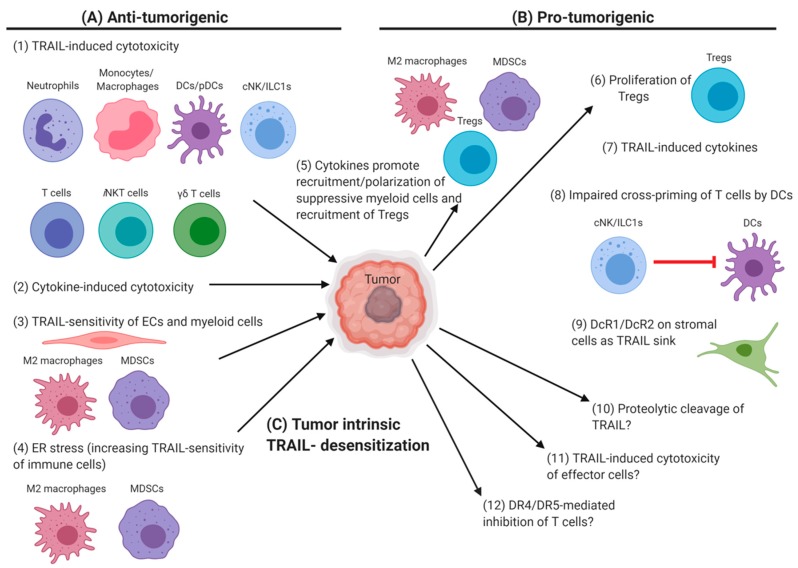
Anti- and pro-tumorigenic activities of TRAIL/DRs in the tumor micro-environment: (**A**) Anti-tumorigenic activities: (1) Several immune cells have been reported to be cytotoxic against tumor cells in an at least partially TRAIL-dependent manner. (2) Besides TRAIL killing the tumor directly, TRAIL-induced cytokines, like TNF, can be cytotoxic for the tumor as well. (3) Endothelial cells (ECs) in the tumor can become TRAIL-sensitive, potentially leading to blood vessels collapse, causing a disruption of the blood supply for the tumor. Similar, pro-tumorigenic M2 macrophages and MDSCs are known to be sensitive towards TRAIL-induced apoptosis. (4) Although, ER stress inside of the tumor is usually associated with pro-tumorigenic effects [329] it can also have an anti-tumorigenic impact. This is mainly due to the increased TRAIL-sensitivity of anti-inflammatory M2 macrophages and MDSCs. (**B**) Pro-tumorigenic activities: (5) TRAIL/DR-induced cytokines produced by the tumor can promote the recruitment and polarization of M2 macrophages and MDSCs, and can promote the recruitment of Tregs. (6) For Tregs it was shown that TRAIL-induced triggering of DR4/DR5 on Tregs can promote their proliferation. (7) TRAIL-induced cytokines, either produced by the tumor or immune cells, can promote tumor survival and proliferation directly or indirectly, e.g., by supporting neo-vascularization. (8) Triggering of DR4/DR5 on DCs by TRAIL^+^ cNK/ILC1s could suppress the cross-priming of tumor-specific T cells by the DCs. (9) Expression of DcR1 and DcR2 on tumor stroma cells can act as sink for TRAIL, reducing its availability to induce tumor cytotoxicity. (10–12) For several other mechanisms, an impact of TRAIL/DRs has not been demonstrated for the tumor microenvironment yet, but their role could be hypothesized based on published data in other contexts. (10) The tumor might promote the proteolytic cleavage of membrane TRAIL from immune cells. (11) TRAIL, either derived from immune cells or from the tumor itself, might promote cytotoxicity of TRAIL-sensitive immune effector cells. (12) TRAIL-mediated triggering of DR4/DR5 on recently activated T cells might inhibit their proliferation. (**C**) To support survival, the tumor can evolve several cell-intrinsic mechanisms to reduce its sensitivity towards TRAIL-induced cytotoxicity. These include the upregulation of anti-apoptotic molecules, the inactivation of signaling molecules, the upregulation of DcR1/DcR2, and changes in DR4/DR5 localization and glycosylation. Alternatively, the tumor can re-purpose the DR4/DR5-signals, for example, to support tumor survival and proliferation, to induce cytokine production by the tumor, and to promote tumor invasion and metastases.

The TRAIL-DR interaction does not only impact the tumor directly, but can also be utilized by the tumor to shape the tumor microenvironment. Treatment of TRAIL-resistant tumor cells with soluble TRAIL or agonistic αDR5-antibodies could promote tumor survival/proliferation [306,313], invasion and metastases [314,315,316,317], and cytokine production [126,316,318,319,320,321]. Such cytokines could induce chemotaxis and recruitment of various myeloid cells [126,318,319,320]. Many mechanisms have been described inside the tumor microenvironment that benefit the tumor [2,322,323,324]. In regard to TRAIL/DRs, several points can be mentioned. It was noted that neutrophils [18,44,45] and DCs [98,102] from the tumor itself or from blood of tumor patients express less TRAIL than cells from control tissue/donors. This reduction might be due to the proteolytic cleavage of TRAIL from the surface, as it was shown for CD178 [325,326,327]. Or the reduction could be a consequence of stimulation, as it was reported, for example, that mouse neutrophils stimulated with IL-6 and G-CSF loose TRAIL expression and their anti-tumor properties [24,25]. Furthermore, DR5-triggering of mouse DCs by TRAIL^+^ cNK/ILC1s reduced their cross-presentation and -priming capacity for tumor-antigens, leading to reduced anti-tumor T cell responses [106]. Finally, expression of DcR1/DcR2 by human stromal cells in the tumor microenvironment could reduce the ligation of DR4/DR5 on tumor cells [328]. All of these mechanisms would reduce the anti-tumor activity of the immune system directly, benefiting the tumor. Moreover, some other TRAIL/DR-dependent mechanisms promote tumor growth indirectly. TRAIL promoted the recruitment and polarization of immune-suppressive M2-like cells [126] and the proliferation of Tregs [190,195,216,217]. Furthermore, it is known that the tumor microenvironment triggers persistent ER stress in infiltrating immune cells and promotes immunosuppressive responses [329]. In regard to TRAIL/DRs, ER stress of myeloid cells has been linked to pro-inflammatory [271] but also anti-tumorigenic activities [30,127]. This latter point illustrates that not all impacts of TRAIL/DRs would benefit TRAIL-resistant tumors. In line with the role of TRAIL/DRs in the elimination of stressed or senescent immune cells, some TRAIL/DR-actions could also suppress tumor growth. For example, the tumor environment induced upregulation of DR5 on mouse MDSCs in vivo for some [127] but not all [28] tumors. Furthermore, mouse and human MDSCs [127] and mouse TAMs [28,63] were shown to be sensitive to TRAIL-induced apoptosis. Consequently, treatment of head and neck cancer patients with agonistic αDR5-antibodies [30] or of tumor-bearing mice with soluble TRAIL [28,62] was able to limit tumor growth to some extent. Additionally, the endothelial cells of tumor-associated blood vessels of some [330] but not all [28] tumor-bearing mice upregulated DR5 and were TRAIL-sensitive, which led to blood vessels collapse and inhibited tumor growth [330]. An overview of the anti- and pro-tumorigenic activities of TRAIL/DRs in the tumor microenvironment is given in Figure 1.

## 5. Conclusions

The original observation that TRAIL preferentially induces apoptosis in tumor cells, while sparing healthy cells, initiated intense research on the development of TRAIL/DR-based anti-cancer therapies. Besides the tumor, innate and adaptive immune cells are a major constituent of the tumor microenvironment, where they can use TRAIL to fight the tumor by inducing apoptosis of tumor cells. However, it becomes increasingly clear that TRAIL/DRs interaction can also directly impact the function of immune cells in many ways. As outlined in this review, many relevant aspects are yet unclear. Therefore, a better understanding of how TRAIL/DRs influence both tumor and immune cells and their interaction within the tumor microenvironment will be essential for the development of successful TRAIL/DR-based anti-cancer therapies.

## Abbreviations

APC	antigen presenting cell
AICD	activation-induced cell death
AML	acute myeloid leukemia
BCR	B cell receptor
BM-DCs	bone marrow-derived dendritic cells
BMDMs	bone-marrow derived macrophages
CAC	colitis-associated colorectal cancer
CBMCs	cord blood-derived mast cells
CCL	C-C motif chemokine ligand
CD	cluster of differentiation
CIA	collagen-induced arthritis
CML	chronic myeloid leukemia
cNK	conventional NK
CNS	central nervous system
CRP	C-reactive protein
CXCR	C-X-C chemokine receptor
DC	dendritic cell
DcR	decoy receptor
DD	death domain
DR	death receptor
DTx	diphtheria toxin
EAE	experimental autoimmune encephalomyelitis
EAT	experimental autoimmune thyroiditis
EoE	eosinophilic esophagitis
Eomes	eomesodermin
ER	endoplasmic reticulum
fMLP	N-Formylmethionyl-leucyl-phenylalanine
GC	germinal center
G-CSF	granulocyte-colony stimulating factor
GM-CSF	granulocyte-macrophage colony-stimulating factor
HBV	hepatitis B virus
HCC	hepatocellular carcinoma
Hsp	heat shock protein
IFN	interferon
Ig	immunoglobulin
IL	interleukin
ILC	innate lymphoid cell
LPS	lipopolysaccharide
MCA	methylcholanthrene
MCMV	murine cytomegalovirus
MDSCs	myeloid derived suppressor cells
mRNA	messenger RNA
MSC	mesenchymal stem cell
*i*NKT	invariant Natural Killer T
NK	Natural Killer
OPG	osteoprotegerin
Pam3C	tri-palmitoyl-S-glyceryl cysteine
pDC	plasmacytoid dendritic cell
PEDF	pigment epithelium derived factor
PHA	phytohemagglutinin
Poly I:C	polyinosinic:polycytidylic acid
RANKL	Receptor activator of nuclear factor kappa-Β ligand
RNA	ribonucleic acid
SDF1	stromal cell-derived factor 1
SLE	systemic lupus erythematosus
TAMs	tumor associated macrophages
TCR	T cell receptor
TGF	transforming growth factor
Th	T helper type
TLR	toll like receptor
TNF	tumor necrosis factor
TNFRSF	TNF receptor superfamily
TRAIL	tumor necrosis factor–related apoptosis–inducing ligand
Treg	regulatory T cell
UPR	unfolded protein response.
